# Exploration of the Vermiculite-Induced Bacterial Community and Co-Network Successions during Sludge–Waste Mushroom Co-Composting

**DOI:** 10.3390/microorganisms12030585

**Published:** 2024-03-15

**Authors:** Zhaojing Yu, Bin Wang, Xiaoyan Wu, Runlan Yu, Li Shen, Xueling Wu, Jiaokun Li, Yuandong Liu, Weimin Zeng

**Affiliations:** 1 School of Minerals Processing and Bioengineering, Central South University, Changsha 410083, China; 185601004@csu.edu.cn (Z.Y.); 215612125@csu.edu.cn (B.W.); yrl715@sina.com (R.Y.); lishen@csu.edu.cn (L.S.); wxl@csu.edu.cn (X.W.); lijiaokun@csu.edu.cn (J.L.); ydoliu@sina.com (Y.L.); 2 School of Resources Environment and Safety Engineering, University of South China, Hengyang 421001, China; wuxiaoyan1005@126.com

**Keywords:** vermiculite additive, co-composting, microbial community, co-network analysis, community function

## Abstract

Vermiculite is a clay mineral with unique physical properties that plays a significant role in plant cultivation, soil remediation, and solid waste management. In this research, we first explored how vermiculite-to-microbe interactions evolved during sludge–waste mushroom residue co-composting. Vermiculite’s addition had a substantial impact on the microbial α and β diversities, significantly changed the microbial community pattern, and strengthened the composting nutrient circulation through the formation of more specialist and generalist species. The microbial community characteristics exhibited common co-networks for resisting composting environment stresses. Vermiculite contributed to enhancing the keystone taxa *Proteobacteria* and *Actinobacteriota* and caused the ecological function network to diversify in the warming and maturation phases, with more complexity and tightness in the thermophilic phase (with super-generalist species existing). The enhanced microbial interactions induced by vermiculite possessed a greater capacity to facilitate the metabolisms of carbohydrates and amino acids and cellulolysis, thereby promoting composting humification, and nitrogen retention in the final compost and composting maturity. These findings are helpful for us to understand the biological process mechanisms of the effect of vermiculite additives on composting and contribute to the establishment of a theoretical framework for enhancing the microbial interactions in composting systems by adding vermiculite in practical applications.

## 1. Introduction

In 2021, the daily treatment capacity of domestic sewage reached 248 million cubic meters, the annual output of sewage sludge exceeded 70 million tons and the annual output of edible fungi reached 37.12 million tons, with waste mushroom residue also exceeding 60 million tons in China. How to safely and economically dispose of these biosolids is currently one of the major environmental issues of concern [1,2]. Waste mushroom residue (WMR) contains a variety of fungal mycelium, decay and cellulose-degrading bacteria, and organic matter, which can be used as a fermentation agent and an excellent inoculant for aerobic sludge composting [3]. Sludge–WMR co-composting for soil remediation and landscaping will hold considerable importance in facilitating the sustainable development of these waste resources.

Vermiculite is a clay mineral that has widespread distribution in China. It possesses notable characteristics such as good air permeability and a high adsorption capacity, causes soil loosening and buffers environmental changes [4], facilitating nutrient utilization by plants, and thus has been widely used in both soiled and soilless cultivation techniques in the field of agriculture [5]. Vermiculite has been used as a viable and easily available bulking agent or an additive in the composting of various biosolid wastes. Liao et al. (1997) reported that vermiculite is an effective additive that can promote nitrogen retention in fish waste composting [6]. Seo evaluated the effect of vermiculite on food waste composting according to the dry basis decomposition rate and demonstrated that the addition of vermiculite increased the decomposition rate of food waste [7]. Tang studied the effect of vermiculite as a leavening agent on the aerobic composting of cow manure and preliminarily evaluated the microbial biomass of composting using the quinone increment (IQC) method, indicating that the IQC is proportional to the mass reduction and cumulative O_2_ consumption [8]. Turan et al. (2009) reported that expanded vermiculite as an effective additive can reduce the amount of volatile organic compounds during the composting of poultry manure [9]. Tang et al. (2010) comparatively characterized the difference in microbial biomass based on a quinone spectroscopy method and the change in bioactivity based on an oxygen consumption method in the composting of three typical biosolid wastes, namely cow manure, food waste, and sewage sludge, under the condition of using vermiculite as a leavening agent and speculated that the microbial biomass rather than the microbial diversity determines the composting activity [10]. Zhang et al. (2012) studied the adsorption behavior of fulvic acid (FA) and humic acid (HA) on kaolinite, montmorillonite, and vermiculite, indicating that at higher FA/HA concentrations, the electron transfer from the aromatic unit of FA to the iron cations induces more FA adsorption into vermiculite than HA adsorption, and HA easily accumulates on the surface of these minerals, while FA is more likely to enter the pores of these minerals and block the pores of the minerals than HA [11]. He et al. (2018) further demonstrated that vermiculite is a suitable additive for food waste composting, as adding vermiculite prolongs the high-temperature phase of composting and accelerates the loss of organic matter and found that vermiculite additives can enhance NH_3_ emissions in the early stage but reduce the NH_3_ release in the later stage, finally facilitating nitrogen retention in the final compost [12]. Pisa et al. (2020) determined the effect of vermiculite inclusion into cattle manure on compost performance and nutrient retention during composting, showing that the inclusion of vermiculite increases the retention of total nitrogen with treatment [13]. Feng and Zhang (2021) demonstrated that vermiculite is a valuable addition to the composting of green waste, and its compost product used as a fertilizer boosts the growth of *Centaurea cyanus* L. [5]. In summary, aerobic composting is an intricate biochemical process [14], and the microbial composition undergoes dynamic changes throughout the composting process [15]. However, to the best of our knowledge, few investigations have reported the effect of vermiculite on the microbial community succession during composting to date, and the biological mechanisms of the nitrogen-holding behavior of vermiculite additives in the composting process remain to be elucidated.

The microbial co-occurrence network is a community-level structural model of species interactions and reflects the functional and structural characteristics of the microbial community in the ecosystem [16,17]. Microbial interactions should be pervasive inside a composting system, and key microorganisms play a vital role in accelerating the transformation of composting matrixes to influence the quality of the compost product [18]. However, most of the previous studies on the effect of additives on composting biodiversity are mostly based on simple diversity indicators, neglecting the importance of complex interactions between species in composting [19,20,21]. Therefore, further study of the evolution of the microbial co-occurrence network under the influence of vermiculite helps reveal the biological process mechanisms of the effect of vermiculite additives on community and function during sludge–WMR co-composting.

The objectives of this study were to (1) investigate the effect of vermiculite additives on microbial community succession during sludge–WMR co-composting and (2) explore the evolutions of the co-occurrence pattern and its function network in the co-composting process. This study contributes to revealing the biological process mechanism of the effect of vermiculite additives on sludge–WMR co-composting.

## 2. Materials and Methods

### 2.1. Composting and Analysis of Physicochemical Property

The dewatered sewage sludge was obtained from the Xinkaipu wastewater treatment plant (Changsha, China). Waste mushroom residue (WMR) was purchased from a nearby farm, and sawdust was collected from a nearby wood processing plant in Changsha. Vermiculite was purchased from Lingshou Mineral Limited Company (Lingshou County, China).

The co-composting experiments were simultaneously carried out in two 50 L stainless steel reactors, as described in our previous work [22]. Briefly, 15 kg of sludge, 12.5 kg of waste mushroom residue, and 2.5 kg sawdust were thoroughly mixed with a shovel to adjust the C/N ratio of the initial mixture of composting raw materials to about 24.5 and the humidity to about 60%. Then, 1% (*w*/*w*) vermiculite was added to the initial mixture and mixed well again. The control group without vermiculite addition and the experimental group with 1% (*w/w*) vermiculite addition were labeled as CK and T1, respectively. Here, to reduce the dilution effect of high-dose vermiculite addition on composting, 1% vermiculite addition was selected to explore the difference in microbial community during the composting with and without vermiculite addition. Each pile was regularly turned. Co-composting experiments lasted for 33 days. Composting samples were collected on days 0, 2, 12, and 33 days, corresponding to the initial, warming, thermophilic, and maturation phases of composting, respectively. These samples were labeled as initial samples, and D2CK, D12CK, and D33CK samples for CK treatment, and D2T1, D12T1, and D33T1 samples for T1 treatment, respectively. Each collected sample was split into three parts [22]. Part one, as a fresh sample, was used to measure moisture content, pH, and electrical conductivity. Part two was freeze-dried, ground, sieved through 0.1 mm sieve, and stored at 4 °C to test TOC, HS, HA, FA, N, P, K. Part three was stored at −80 °C for extracting DNA and a sequencing analysis [22,23]. All composting physicochemical properties were analyzed according to these methods described in our previous work [22,23] (Table S1). All samples were tested in triplicate.

### 2.2. DNA Extraction, High-Throughput Sequencing

DNA was extracted from the composting sample, and high-throughput sequencing was analyzed as described in our previous work [20]. Briefly, the extraction of total genomic DNA was performed using a FastDNA^®^ Spin Kit for Soil (MP Biomedicals, Santa Ana, CA, USA), and followed the workflows using PCR (Thermos Fisher Scientific, Waltham, MA, USA), purity, and concentration (GeneJET, Thermos Fisher Scientific, Waltham, MA, USA). High-throughput sequencing was carried out on the NovaSeq6000 platform of Magi gene Co., Ltd. (Guangzhou, China) through the amplification of the V3–V4 region of bacterial 16S rRNA using 515F/907 R(5′GTGCCAGCMGCCGCGG-3′/5′CCGTCAATTCMTTTRAGTTT-3′). A bioinformatics analysis of 16SrRNA gene sequences was carried out using QIIME2. Raw data were uploaded to NCBI SRA database (serial number: PRJNA1065105).

### 2.3. Quality Control and Statistical Analysis

The raw data obtained from the Illumina platform were filtered using FASTP (version 0.19.6, https://github.com/OpenGene/fas, accessed on 17 March 2023) to obtain clean reads. The clean reads obtained after filtering were then used for assembly analysis. The obtained clean reads were merged into tags with a minimum overlap of 10 bp and a maximum mismatch rate of 2% using FLSAH (version 1.2.11, https://ccb.jhu.edu/software/FLAS, accessed on 17 March 2023) and filtering the tags. The clean tags were clustered into OTUs with a similarity of ≥97% using Upraise (version 7.1, http://drive5.com/uparse/, accessed on 17 March 2023). After filtering the tag chimerism using USEARCH (version 11, http://www.drive5.com/usearch/, accessed on 17 March 2023) for tag chimeric examination, the obtained effective tags were used for OUTs abundance statistics and other subsequent analyses.

All downstream community data were analyzed using RStudio software (R. version 4.2.3) [24]. The “ggplot2” package (version 3.5.0) was used to plot a variety of graphs. The “microeco” package (version 1.5.0) was used to analyze community α-diversity and β-diversity. The “metacoder” package (version 0.3.7) was used to draw species classification trees. Generalist and specialist analyses were performed using the “vegan” package (version 2.6-4). The “igraph” package (version 1.4.3) was used for a co-network analysis and generated using Gephi (version 0.10.1). The screening criteria for the network analysis were *p* < 0.05|r > 0.8, based on Spearman correlation. The modular topology was further studied using the “Hmisc” package (version 5.1-1) and the “zi-pi. r” programs to explore the role of the community in the network. PICRUSt2 (version 2.2.0, https://github.com/picrust/picrust2, accessed on 20 April 2023) was used as a KEGG functional gene prediction, and Functional Annotation of Prokaryotic Taxa (FAPROTAX) was used as a functional annotation. All the data were statistically tested at a significance level of 0.05 [25].

## 3. Results and Discussion

### 3.1. α and β Diversities

A dataset of 954580 bacterial 16S rRNA sequences of excellent quality was obtained using rigorous quality control and filtering processes (Table S2). These sequences had an average length of 416 base pairs. Based on 97% sequence similarity, 3521 bacterial OTUs were obtained. All rarefaction curves for bacterial sequences had reached a plateau, indicating that the depth of bacterial sequencing had reached a saturation point. All the coverage indices were above 0.99 (Figure S1). These results indicated that the quantity of sequencing data obtained was sufficient for subsequent analyses.

**Figure 1:**
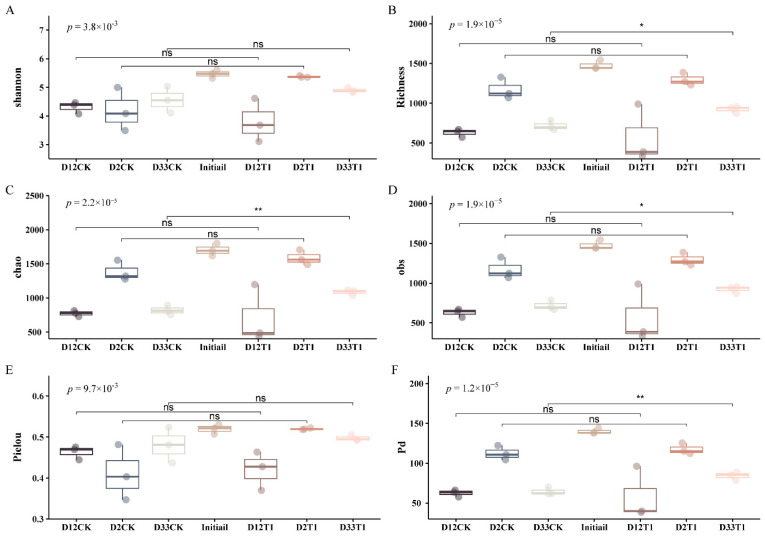
Alpha diversity of the microbial community during composting. (**A**) Shannon index. (**B**) Richness. (**C**) Chao index. (**D**) obs (observed species). (**E**) Pielou index. (**F**) Pd (Phylogenetic diversity) index. (ns means not statistically difference, * means the level of difference was at 0.05, ** means the level of difference was at 0.01.).

Compared to the initial mixture, the biodiversity indices of CK and T1 treatments during whole composting exhibited a statistically significant decrease, as shown in Figure 1. As composting progressed, the biodiversity indices showed a trend of first decreasing and then increasing from the warming phase to the thermophilic phase to the maturity phase. Microbial richness and diversity were their lowest in the thermophilic phase. This may be due to the high-temperature composting which efficiently eliminated the thermo-intolerant bacteria [26] and selectively promoted the growth of microorganisms adapted to the composting environment [27]. Compared to CK, microbial richness and diversity of T1 (1% vermiculite treatment) were higher at the warming and maturity phases, but lower at the thermophilic phase. This difference resulted from the adsorption and catalytic effects of vermiculite [22,28]. These indices indicated that vermiculite addition exerted a significant influence on the diversity of the microbial community, and enhanced the abundance and balance of microbial species involved in the co-composting process (Table S3).

**Figure 2:**
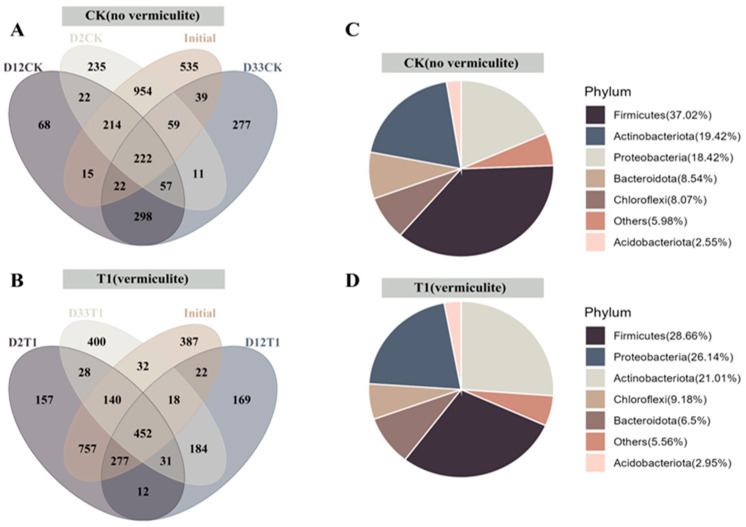
Community structure composition of composting. (**A**,**B**) Venn diagrams of OTUs in different composting phases of CK and T1, respectively; (**C**,**D**) communities at phylum level of CK and T1, respectively. (OTUs means operational taxonomic units, one OTU can represent one bacterial classification).

**Figure 3:**
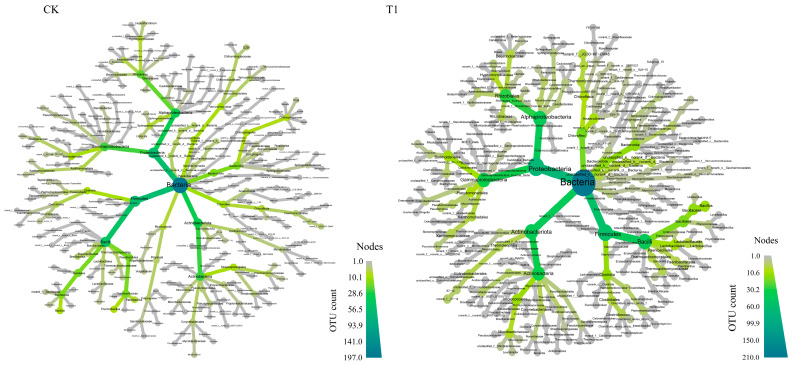
Species classification trees in CK and T1 treatments.

To understand the resemblance and specificity of the species distribution of microbial communities in T1 and CK treatments, a β-diversity analysis was conducted to gain insights into species variation [29]. As depicted in Figure 2A,B, the shared OTUs in different composting phases of CK and T1 were 222, and 452, respectively, and the difference between CK and T1 groups was 230, and the latter was much greater than the former. This suggested that vermiculite improves the composting microenvironment to promote the growth of bacteria responsible for organic matter metabolism during the co-composting process. As depicted in Figure 2C,D, the predominant phyla in CK and T1 treatments all were *Firmicutes*, *Actinobacteria*, and *Proteobacteria*, which were consistent with most composting studies [23,30], and their relative abundances were 37.02%,19.42%, 18.42% for CK, and 28.66%, 26.14%, 21.01% for T1, respectively. The vermiculite addition did greatly change the relative abundances of predominant species of core community during the co-composting. Compared to CK, *Proteobacteria* and *Actinobacteria* were higher and *Firmicutes* were lower in T1. Vermiculite enhanced the ability of *Actinobacteria* and *Proteobacteria* to resist unfavorable conditions, facilitating the breakdown of lignocellulose [31], organic matter decomposition, and nitrogen cycling [32]. The species classification tree analysis (Figure 3) demonstrated that T1 more than CK had greater trunks and branch abundances, and vermiculite significantly enhanced the abundance and balance of the microbial species involved in the co-composting process, and especially promoted core species *Alphaproteobacteria*, *Gammaproteobacteria*, *Actinobacteria*, and *Bacilli* in the co-composting system.

**Figure 4:**
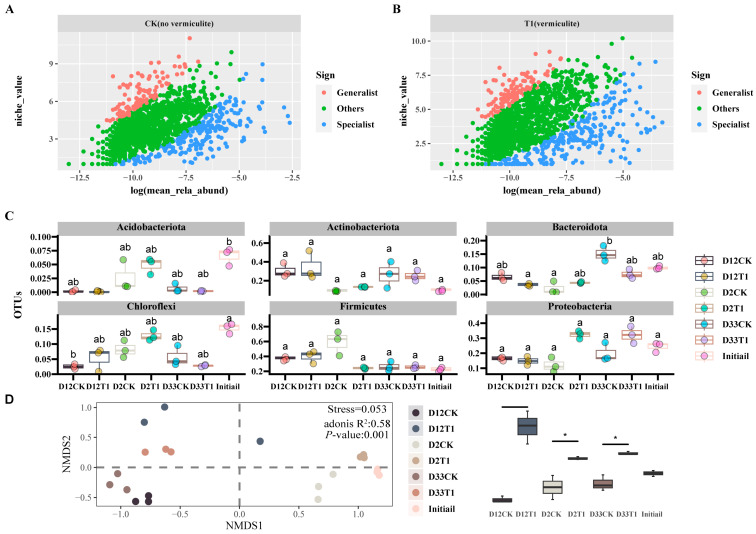
The evolution of microbial species with the composting process in CK and T1 treatments, respectively. (**A**,**B**) Specialist and generalist species; (**C**) analysis of species differences at the phylum level (Top six); (**D**) NMDS analysis of bacterial communities at different composting periods. (NMDS means non-metric multidimensional scaling, * means the level of difference was at 0.05. Letter differences indicate whether there is a difference between the two treatments. Lowercase letters represent the statistical level at 0.05.).

In specific habitats, two distinct types coexisted in bacterial communities, generalist and specialist microbes [33]. There are significant distinctions between the two types of species in terms of their reactions to resource utilization, environmental adaptation, and microbial interactions. As seen from Figure 4A,B, compared to CK, T1 had much more generalist and specialist species, and the generalist species with medium–high niches were more aggregated, and the specialist species with low, medium, and high niches were more dispersed. These results indicated that T1 over CK was significantly stronger in nutrient cycling such as of carbon and nitrogen [34,35]. *Actinobacteria* are widely recognized as the main species responsible for the degradation of recalcitrant organic compound lignin in composting [14]. *Firmicutes* are strongly capable of tolerating environmental stresses and can produce several enzymes, including protease, cellulase, and hemicellulose enzymes [29]. *Proteobacteria* have an excellent ability to decompose organic matter, such as macromolecular protein, starch, and other organic matter [23]. Figure 4B shows the changes of predominant microbes in different composting stages. *Actinobacteria* in T1 had a slightly higher abundance than CK at the warming and thermophilic phases. *Firmicutes* in T1 over CK had very low abundance at the warming phase, but there was almost no difference in *Firmicutes* abundance between CK and T1 at thermophilic and maturity phases, while *Proteobacteria* in T1 had much higher abundance than CK at the warming and maturity phases. Thus, the water-absorbing property of vermiculite was not conducive to the growth of *Firmicutes* in the early stage of composting, and had almost no influence on *Firmicutes* abundances in thermophilic and maturity stages, while the loose, porous, and absorption properties of vermiculite were conducive to promoting the growth of *proteobacteria* and *Actinobacteria* to break down the composting of organic matter [36]. For the CK group, dominant genera mainly were *Bacillus* (20.38~42.32%), *Ureibacillus* (6.72~20.07%) in D2CK, and *Pseudoxanthomonas* (3.51~4.82%), *Thermobacillus* (5.07~5.20%), *Saccharomonospora* (2.10~2.93%), and *Thermotunica* (11.2~18.95%) in D12CK, and the above genera were still the main dominant species in D33CK. For group T1, the dominant genera were *Romboutsia* (4.31~5.5%), *Acinetobacter* (4.95~6.28%) in D2T1, and *Bacillus* (13.58~22.89%), *Pseudoxanthomonas* (4.12~9.62%), *Saccharomonospora* (4.25~10.7%), *Streptomyces* (3.63~4.23%), *Ochrobactrum* (2.11~3.87%), *Nonomuraea* (0.62~10.7%), *Thermotunica* (1.21~3.87%) in D12T1, and the above genera remained dominant in D33T1, especially *Bacillus* (13.14~16.04%), *Thermotunica* (4.21~7.11%) and *Streptomyces* (2.10~2.32%). The above genera usually play an important role in composting [22]. The genera influenced significantly by vermiculite were mainly *Ureibacillus*, *Romboutsia*, *Acinetobacter*, *Streptomyces*, and *Ochrobactrum*.

A non-metric multidimensional scaling (NMDS) analysis was performed on the samples at different composting phases, with the Bray–Curtis distance serving as the algorithm’s foundation, as shown in Figure 4C. Using PERANOVA (“Vegan” version 2.6-4), NMDS had superior results for community differentiation (stress = 0.053 < 0.2|*p* = 0.001). The bacterial community structure was mainly clustered into three groups. On the second day of co-composting, microbial communities had very small distinctions between CK and T1. However, as composting progressed, especially at the thermophilic phase, the microbial communities with and without vermiculite exhibited distinct separation along the 1- and 2-axes of the NMDS. Therefore, the addition of vermiculite significantly changed the community succession pattern and had an advantageous effect on sludge–WMR co-composting [16,24].

### 3.2. Analysis of Co-Network and Modular Topology

**Figure 5:**
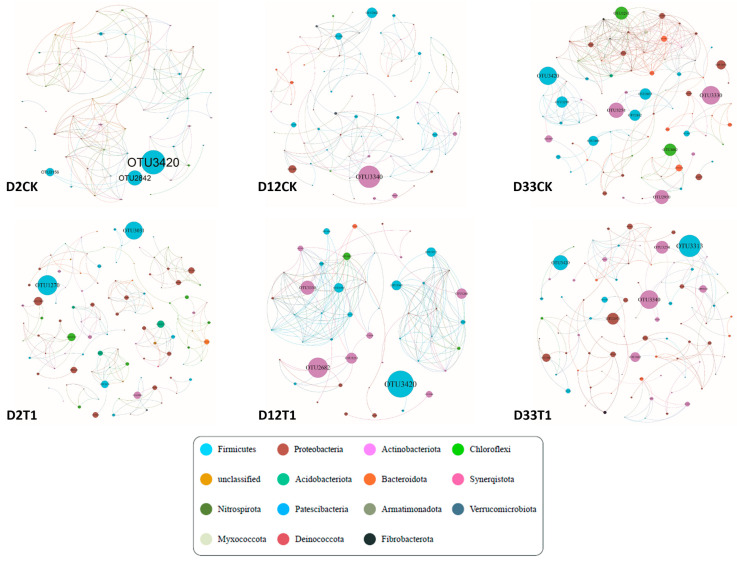
The evolution of microbial correlation network with composting process. The node’s size denotes the abundance of species, the node’s color represents unique phyla, the red line represents positive correlation, and the blue line represents negative correlation.

A co-network analysis was conducted to assess the microbial interaction during composting, as shown in Figure 5. The screening criterion utilized was a significance level of *p* < 0.05 and a correlation coefficient (R) > 0.8. The average path lengths (GD) observed in CK and T1 varied between 1.911 and 3.917, and 1.407 and 2.345, respectively (Table S4). The microbial community characteristics exhibited commonly small-world co-networks. The modularity (M) of all networks was greater than 0.4, and the average clustering coefficients and modularity were significantly higher than those of the random network generated with the same number of nodes and connecting edges (replicate = 10,000). The constructed microbial ecological networks accurately reflected the community dynamics and the interrelationships among different species in the co-composting process. As composting progressed, the number of nodes and edges increased from 60 to 84 and 160 to 286 in the CK group, respectively; however, it decreased from 108 to 86 and 196 to 131 in the T1 group, respectively. The modularity of T1 was 0.291, higher than that of CK in the maturation period. Thus, vermiculite made the co-network tighter, and microbial communities more stable [14,37]. A co-network analysis revealed that the related microbial genera in CK belonged to *Firmicutes* (25~35%), *Proteobacteria* (25~26.25%), *Actinobacteria* (1.19~15%), and *Bacteroidetes* (3.33~17.86%), and the related microbial genera in T1 belonged to *Firmicutes* (18.75~43.75%), *Proteobacteria* (29.17~34.88%), *Actinobacteria* (15.18~20.93%), and *Bacteroidetes* (2.08~10.47%) (Tables S5 and S6). These bacteria played a crucial role in the degradation of both organic matter and lignin in co-composting [1,37,38]. Thus, the change of specialized metabolic functions of keystone taxa by adapting to the effects of vermiculite and composting processes sustained the stability of the composting community function network [37].

**Figure 6:**
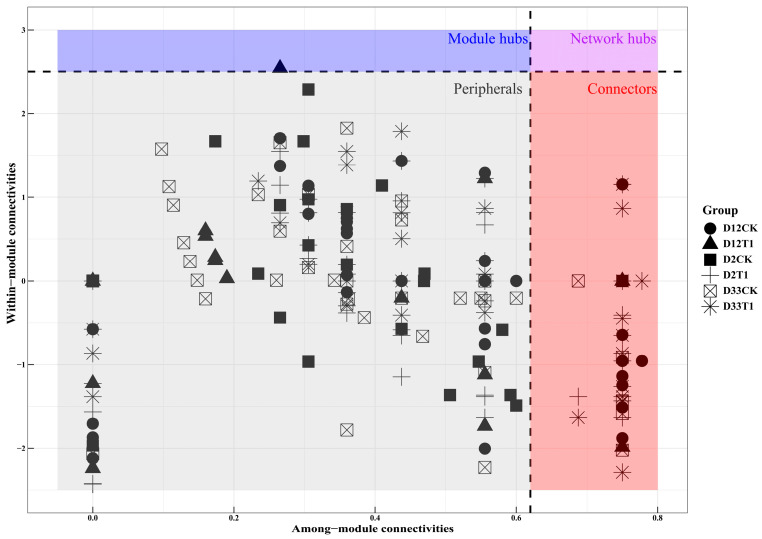
The evolution of microbial co-network with composting process based on modular topology analysis.

Different nodes play different topological roles in co-network, and module topology analysis is important for the identification of key taxa in the composting process [37]. In the module topology of the co-network, peripherals may be specialist species, and connectors and module hubs are closer to generalist species that play an important role in network topology as keystone species [36,39]. As seen in Figure 6, almost all keystone species belonged to connectors and module hubs during co-composting. In the CK group, a total of 24 (5 + 12 + 7) connectors were detected within the ecological networks, and 5, 12, 7 connectors corresponded to D2CK, D12CK, D33CK, respectively, and the predominant taxa identified among these important species were associated with *Firmicutes* (16), *Bacteroidota* (4), *Actinobacteriota* (2), *Verrucomicrobiota* (2), and *Proteobacteria* (1). In the T1 group, a total of 52 (18 + 3 + 31) connectors and one module hub were detected within the ecological networks, and 18, 3, and 31 connectors corresponded to D2T1, D12T1, D33T1, respectively, and one Module hub existed in D12T1, and the predominant taxa consisted of *Proteobacteria* (18), *Firmicutes* (16), *Actinobacteriota* (10), *Bacteroidota* (4), *Verrucomicrobiota* (2), *Chloroflexi* (2), *Cyanobacteria* (1). These microorganisms that played the role of connectors were dominated by *Firmicutes*, *Proteobacteria*, *Bacteroidota*, and *Nitrospirota*. In terms of the number of connectors, D2T1 > D2CK, D33T1 > D33CK, and although D12CK > D12T1, one module hub (super-generalist) existed in D12T1. Obviously, vermiculite contributed to a great increase in potential functional *Proteobacteria* and *Actinobacteriota* in T1, while *Firmicutes* was a common keystone species in CK and T1; at the same time, vermiculite made the ecological function network diversified at the waring and maturation phases, and complicated and compacted at the thermophilic phase. Thereby, vermiculite enhanced community functions by strengthening the co-network of keystone taxa to adapt to the change in the composting environment stress [18], promoting substrate transformation and humification of the co-composting.

### 3.3. Function Analysis of Metabolism Networks

**Figure 7:**
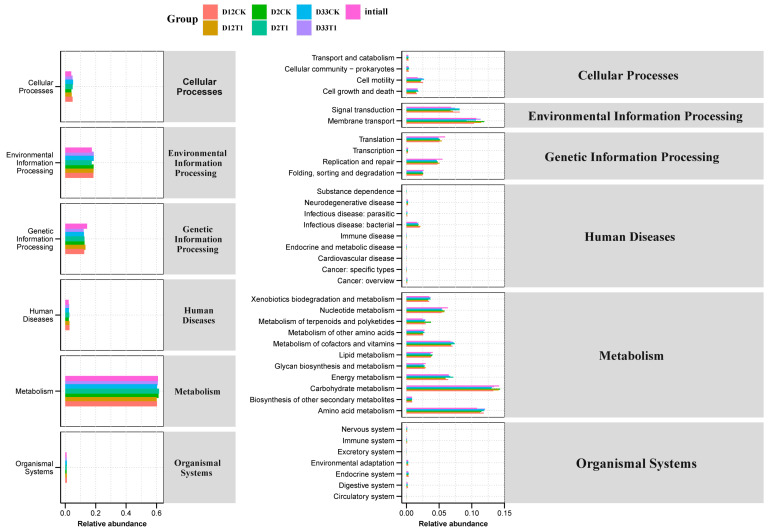
Analysis of primary metabolic pathway and secondary metabolic pathways.

The potential functional and metabolic pathways of microbial communities were investigated using KEGG analysis during co-composting [40], as shown in Figure 7. Regardless of whether vermiculite was added or not, the relative abundances of functional genes related to metabolism pathways, environmental information processing, genetic information processing, and cellular processes accounted for 60.09–61.64%, 17.3–18.74%, 12.07–14.32%, and 3.93–5.08%, respectively. Among them, the abundance of functional genes related to metabolic pathways was the highest (>60%). The effect of vermiculite did not seem to be significant from the point of view of the primary metabolic pathway. The secondary metabolic pathways showed that the functional genes with high abundance were related to carbohydrate metabolism, amino acid metabolism, cell membrane transport, and signal transduction in turn. They were notably higher in T1 than CK. This was due to the fact that vermiculite promoted *Bacillus*, *Pseudoxanthomonas*, *Saccharomonospora*, *Streptomyces*, *Ochrobactrum*, and *Nonomuraea,* mentioned above. As demonstrated by Yin et al. (2019), the breakdown of lignocellulose is positively influenced by carbohydrate metabolism, and composting maturation is closely linked to the metabolism of amino acids [41]. So, the KEGG metabolic pathway analysis indicated that vermiculite promoted the degradation of organic matter (carbohydrate and amino acid metabolisms) in composting and composting maturity. This is consistent with the results of our determination of the physicochemical properties of composting.

**Figure 8:**
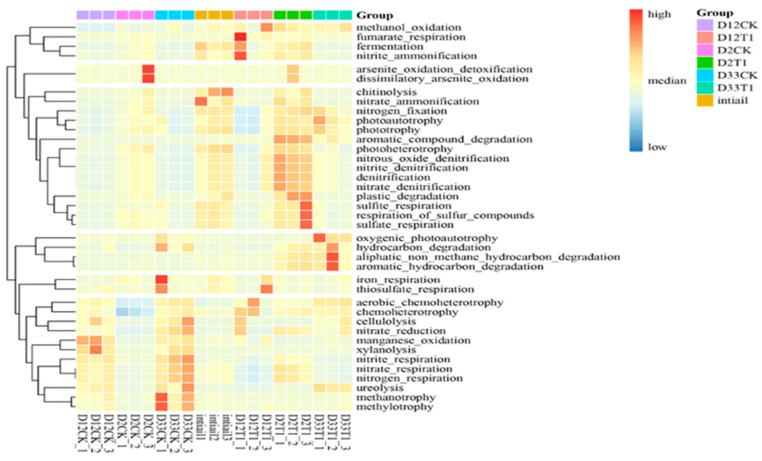
Functional gene annotation based on FAPROTAX.

FAPROTAX (Functional Annotation of Prokaryotic Taxa) over KEGG is more suitable for annotating functional genes in environmental samples [42], as shown in Figure 8. On the second day of co-composting (D2 sample), the abundances of genes related to the degradation of aromatic, aliphatic hydrocarbon compounds, plastic, chemoheterotrophy, chitinolysis were significantly higher in T1 than in CK, which indicated that vermiculite addition promoted the degradation of cellulose substances and facilitated a quick start to co-composting. On the 12th day of co-composting (D12 sample), the abundance of genes related to cellulolysis was significantly higher in T1 than CK. On the 33th day of co-composting, the abundances of genes related to the degradation of aromatic, aliphatic, and hydrocarbon matters were high in T1, but the abundances of genes related to the methanotrophy, methylotrophy, cellulolysis, xylanolysis were high in CK. These results indicated that vermiculite addition was conducive to the decomposition and utilization of composting organic matter, enhanced cellulolysis, and shortened the composting maturity period, thereby promoting the humification of co-composting. This was consistent with our determination results of the physicochemical properties of composting (relative experimental data are not provided here). In addition, compared to CK, the abundance of genes related to nitrogen metabolisms in T1 was high at the warming phase, but very low at the maturity phase (Figure S2). It indicated that vermiculite promoted the release of NH_3_ in the early stage of composting, but significantly reduced the release of NH_3_ in the middle-to-late stage. Interestingly, vermiculite promoted nitrogen retention in the final compost. This also was consistent with our determination results of the physicochemical properties of the composting. This result also very well explained the reported behavior of vermiculite affecting ammonia release in food waste composting [12]. The differences in microbial metabolisms between T1 and CK were mainly due to the vermiculite-enhanced *Bacillus*, *Pseudoxanthomonas*, *Saccharomonospora*, *Streptomyces*, *Ochrobactrum*, and *Nonomuraea*. However, the biological mechanisms of nitrogen metabolism and nitrogen retention under the influence of vermiculite remain to be studied in depth.

## 4. Conclusions

In this research, we first investigated how a vermiculite additive influenced the microbial community succession and its function network evolution in the co-composting process of sewage sludge and waste mushroom residue. Vermiculite had a substantial impact on microbial α and β diversities in the composting process, significantly changed community patterns, and strengthened composting nutrient circulation using the formation of more specialist and generalist species. Microbial community characteristics exhibited commonly small-world co-networks to maximize the microbial synergistic effect and adapt to environmental change during composting. Vermiculite contributed to great increase in potential functional species *Bacillus*, *Pseudoxanthomonas*, *Saccharomonospora*, *Streptomyces*, *Ochrobactrum*, and *Nonomuraea*, and made the ecological function network more diversified at the warming and maturation phases, and complicated and compacted at the thermophilic phase (super-generalist existing). The enhanced microbial interactions induced by vermiculite possessed greater capacity to facilitate the metabolisms of carbohydrates, amino acids, and cellulolysis, thereby promoting the composting humification, and nitrogen retention in the final compost and composting maturity. These findings are helpful for us to understand the biological mechanism of the effect of a vermiculite additive in the composting process, and contribute to the establishment of a theoretical framework for enhancing microbial interactions in composting systems by adding vermiculite in practical applications.

## Data Availability

Data are available on request.

## Supplementary Materials

The following supporting information can be downloaded at: https://www.mdpi.com/article/10.3390/microorganisms12030585/s1, Figure S1: Sobs index and Coverage; Figure S2: The variation of temperature, humus, N, P, K during composting, (a) Variation of pile temperature with composting time, (b) Variation of pile temperature with composting time, (c) Variation of pile temperature with composting time. CK-0 CK-F: initial and final composting without vermiculite, respectively; T1-Z0 and T1-ZF: initial and final composting with vermiculite, respectively; Table S1: Physical and chemical properties of raw materials for composting; Table S2: Effective sequence number of composting in different periods; Table S3: Changes in the community α-diversity; Table S4: Topological properties of the empirical MENs of microbial communities and their associated random MENs; Table S5: Percentage of composting at phylum level at different treatments. Table S6: Percentage of composting at genus level at different treatments.
