# Supplementary material for: Exploration of the Vermiculite-Induced Bacterial Community and Co-Network Successions during Sludge–Waste Mushroom Co-Composting
Source: Microorganisms. 2024 Mar 15;12(3):585. doi: 10.3390/microorganisms12030585 (PMC10976085; doi:10.3390/microorganisms12030585)
Supplement: Supplementary file 1 [file microorganisms-12-00585-s001.zip › microorganisms-2881283-supplementary.pdf]

Supplementary material

Influence of vermiculite on microbial succession processes during sludge-waste mushroom co-composting

Zhaojing Yu a1, Bin Wang a1, Xiaoyan Wu c, Runlan Yu a,b, Li Shen a,b, Xueling Wu a,b, Jiaokun Li a,b, Yuandong Liu a,b, Weimin Zeng a,b*

a School of Minerals Processing and Bioengineering, Central South University, Changsha, 410083, China

b Key Laboratory of Biometallurgy, Ministry of Education, Changsha, 410083, China

c School of Resources Environment and Safety Engineering, University of South China, Hengyang 421001, China

* Corresponding author: Weimin Zeng, E-mail: zengweimin1024@126.com;

Supplementary material includes:

- 6 Tables
- 2 Figures

Supplementary Table S1

Physical and chemical properties of raw materials for composting

	Sludge	Waste mushroom	Sawdust	Vermiculite
TOC(g/kg)	254.70±1.5	459.9±3.5	574.3±2.3	ND
TN(g/kg)	41.82±0.90	10.80±0.69	5.90±0.10	5.30±0.3
C/N	6.09	42.58	97.34	ND
pH	7.02±0.03	8.26±0.04	6.51 ± 0.03	7.32±0
MC (%)	80.16±0.08	17.83±0.06	9.67±0.04	1.2±0.21

TOC: total organic carbon; TN: total nitrogen; MC: moisture content

Supplementary Table S2

Effective sequence number of composting in different periods

Samples	Sequences	Bases	Average Length
Initial1	41456	17236501	415.78
Initial2	50126	20771417	414.38

Initial3	42358	17560357	414.57
D2CK_1	43252	18281834	422.68
D2CK_2	39620	16649564	420.23
D2CK_3	41415	17269858	417.00
D2T1_1	41912	17332119	413.53
D2T1_2	41463	17124160	413.00
D2T1_3	51470	21255672	412.97
D12CK_1	43009	18050540	419.69
D12CK_2	49510	20715470	418.41
D12CK_3	37762	15835122	419.34
D12T1_1	52836	22203950	420.24
D12T1_2	49646	20681088	416.57
D12T1_3	39363	16357958	415.57
D33CK_1	46543	19465972	418.24
D33CK_2	43316	18066638	417.09
D33CK_3	49690	20849177	419.58
D33T1_1	45562	18953697	415.00
D33T1_2	59958	24292104	415.64
D33T1_3	44313	18431876	415.95

Supplementary Figure S1

Sobs index and Coverage

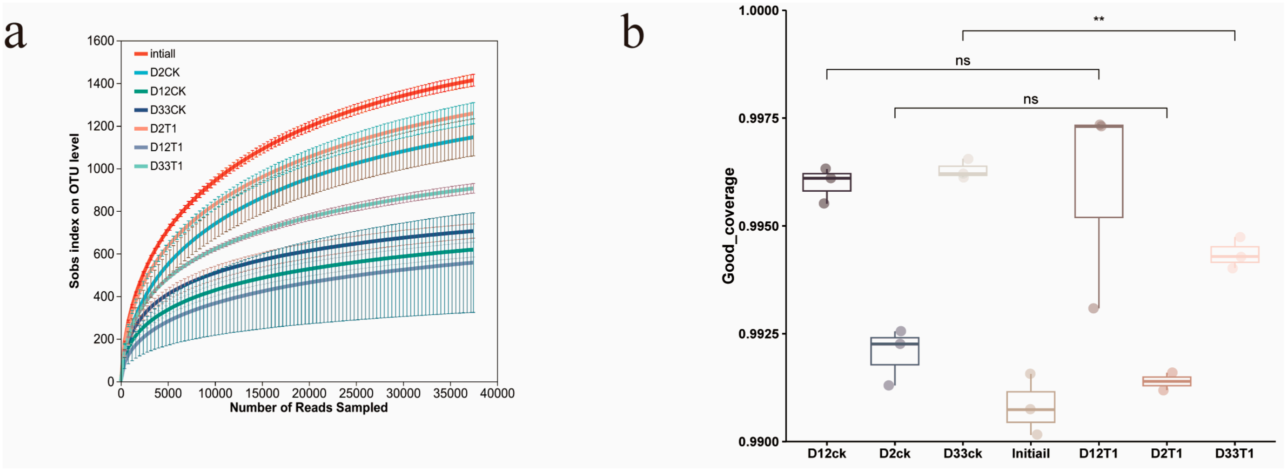

Table S3

Supplementary table for 3.1 " **Changes in the community α-diversity** "

Alpha diversity index

	Shannon	Richness	Chao	Obs	Pielou	Phylogenetic diversity
Initial	5.47^{a}(0.15)	1476^{a}(59.81)	1704^{a}(92.9)	1476^{a}(59.81)	0.52^{a}(0.01)	91.42^{a}(32.56)
D2CK	4.19^{cd}(0.76)	1173^{bc}(136.64)	1383^{ab}(148.94)	1173^{bc}(136.64)	0.41^{c}(0.07)	112.56^{b}(9.15)
D2T1	5.37^{ab}(0.03)	1296^{ab}(81.84)	1585^{a}(110.62)	1296^{ab}(81.84)	0.52^{a}(0)	117.70^{ab}(7.04)
D12CK	4.31^{cd}(0.21)	629.67^{e}(51.98)	772.37^{cd}(43.41)	629.67^{e}(51.98)	0.46^{abc}(0.02)	62.47^{cd}(4.37)
D12T1	3.80^{d}(0.76)	572^{e}(362.93)	707.23^{d}(425.28)	572^{e}(362.93)	0.42^{bc}(0.05)	58.22^{d}(33)
D33CK	4.56^{bcd}(0.47)	717.67^{de}(60.93)	819.49^{cd}(69.87)	717.67^{de}(60.93)	0.48^{ab}(0.04)	64.75^{cd}(4.78)
D33T1	4.9^{abc}(0.09)	923.33^{cd}(45.79)	1084^{bc}(45.39)	923.33^{cd}(45.79)	0.50^{a}(0.01)	84.27^{c}(5.22)

obs (observable OUT), abcde means P values were significant at 0.05 level (ANOVA, post hoc test: Duncan)

Table S4

Supplementary table for 3.3 " **Interaction network of microbial community and modular topology** "

Topological properties of the empirical MENs of microbial communities and their associated random MENs

	**Empirical network**						**Random network (n=10,000)**		
	Total nodes	Total edges	Average degree (avgK)	Average clustering coefficient (avgCC)	Average path distance (GD)	Modularity (M)	Average clustering coefficient (avgCC)±SD	Average path distance (GD)± SD	Modularity(M) ±SD
D2CK	60	160	5.333	0.825	3.917	0.707	0.256±0.005	1.75±0.001	0.135±0.006
D12CK	80	140	3.5	0.758	1.911	0.874	0.043±0.016	3.508±0.092	0.484±0.018
D33CK	84	286	6.81	0.796	2.802	0.597	0.082±0.011	2.496±0.0151	0.316±0.012
D2T1	108	196	3.63	0.839	1.899	0.915	0.034±0.011	3.67±0.075	0.492±0.015
D12T1	48	172	7.167	0.896	1.407	0.627	0.15±0.017	2.14±0.0167	0.271±0.015
D33T1	86	131	3.407	0.796	2.345	0.888	0.035±0.016	3.91±0.1367	0.533±0.019

Table S5

Supplementary table for 3.3 " **Interaction network of microbial community and modular topology** "

Percentage of composting at phylum level at different treatments

**Phylum (%)**	**D2CK**	**D12CK**	**D33CK**	**D2T1**	**D12T1**	**D33T1**
Firmicutes	26.67	35	25	18.75	43.75	23.26
Proteobacteria	25	26.25	26.19	34.82	29.17	34.88
Actinobacteriota	15	13.75	1.19	15.18	20.83	20.93
Chloroflexi	13.33	5	7.14	12.5	4.17	3.49
Unclassified	6.67	--	--	5.36	--	--
Acidobacteriota	5	1.25	11.9	4.46	--	--
Bacteroidota	3.33	8.75	17.86	4.46	2.08	10.47
Synerqistota	1.67	--	--	0.89	--	--
Nitrospirota	1.67	--	--	0.89	--	--
Patescibacteria	1.67	--	--	0.89	--	--
Armatimonadota	--	--	--	0.89	--	--
Verrucomicrobiota	--	6.25	2.38	0.89	--	1.16
Myxococcota	--	2.5	5.95	--	--	2.33
Deinococcota	--	1.25	1.19	--	--	--
Fibrobacterota	--	--	1.19	--	--	--

Table S6

Supplementary table for 3.3 " **Interaction network of microbial community and modular topology** "

Percentage of composting at genus level at different treatments (Top 15)

Treatment	Genus	Percentage (%)	Treatment	Genus	Percentage (%)
D2CK	*Bacillus*	5.71	D2T1	*Unclassified Bacteria*	5.36
	Unclassified Bacteria	5.71		*Acinetobacter*	4.46
	Saccharimonadales	2.86		*Hyphomicrobium*	2.68
	67-14	2.86		*SBR1031*	2.68
	Lactobacillus	2.86		*Caldilineaceae*	2.68
	Hyphomicrobium	2.86		*JG30-KF-CM45*	2.68
	1-20	2.86		*Pseudoxanthomonas*	1.79
	SC-1-84	2.86		*67-14*	1.79
	Microtrichales	2.86		*Rhodobacteraceae*	1.79
	SBR1031	2.86		*Microtrichales*	1.79
	Caldilineaceae	2.86		*BD 1-7 clade*	0.89
	JG30-KF-CM45	2.86		*Nitrosomonas*	0.89
	C10-SB1A	2.86		*Bacillus*	0.89
	BD1-7 clade	1.43		*Proteiniclasticum*	0.89
	Acidaminococcaceae	1.43		*Nitrospira*	0.89
D12CK	*Thermobacillus*	5.56	D12T1	*Bacillus*	10.34
	Bacillus	4.44		*Pseudoxanthomonas*	3.45
	Brevibacillus	4.44		*Bordetella*	3.45
	DSSF69	3.33		*Lactobacillus*	3.45
	Vulgatibater	2.22		*Ureibacillus*	3.45
	Verrucomicrobiaceae	2.22		*Pseudomonas*	3.45
	Thermoactinomyces	2.22		*Thermoactiomyces*	3.45
	Tepidimicrobium	2.22		*Cellulosmicrobium*	1.72
	Persicitalea	2.22		*Microbacterium*	1.72
	Planifilum	2.22		*Enterococcus*	1.72

	Thermobispora	2.22		*Pseudoclavibacter*	1.72
	Paenibacillus	2.22		*Bradyrhizobium*	1.72
	Thermomonospora	1.11		*Streptomyces*	1.72
	Parasegetibacter	1.11		*Niabella*	1.72
	Sporocytophaga	1.11		*67-14*	1.72
D33CK	*Bacillus*	5.43	D33T1	*Bacillus*	6.38
	Thermobacillus	3.26		*JG30-KF-CM45*	4.26
	Paenibacillus	3.26		*Devosia*	3.19
	Roseiflexaceae	2.17		*Pseudoxanthomonas*	2.13
	Puia	2.17		*Ureibacillus*	2.13
	Chryseobacterium	2.17		*Pseudomonas*	2.13
	Bordetella	2.17		*Microbacterium*	2.13
	Rhizobiaceae	2.17		*Sphingobium*	2.13
	JG30-KF-CM45	2.17		*Rhizobiaceae*	2.13
	Devosia	2.17		*Paenibacillus*	2.13
	Flavobacterium	2.17		*Thermobacillus*	1.06
	Pseudopedobacter	1.09		*Cellulosimicrobium*	1.06
	Cellulosimicrobium	1.09		*Sporocytophaga*	1.06
	Pseudoxanthomonas	1.09		*Bradyrhizobium*	1.06
	Geobacillus	1.09		*Streptomyces*	1.06

Supplementary Figure S2

Figure S2 The variation of temperature，humus，N、P、K during composting.

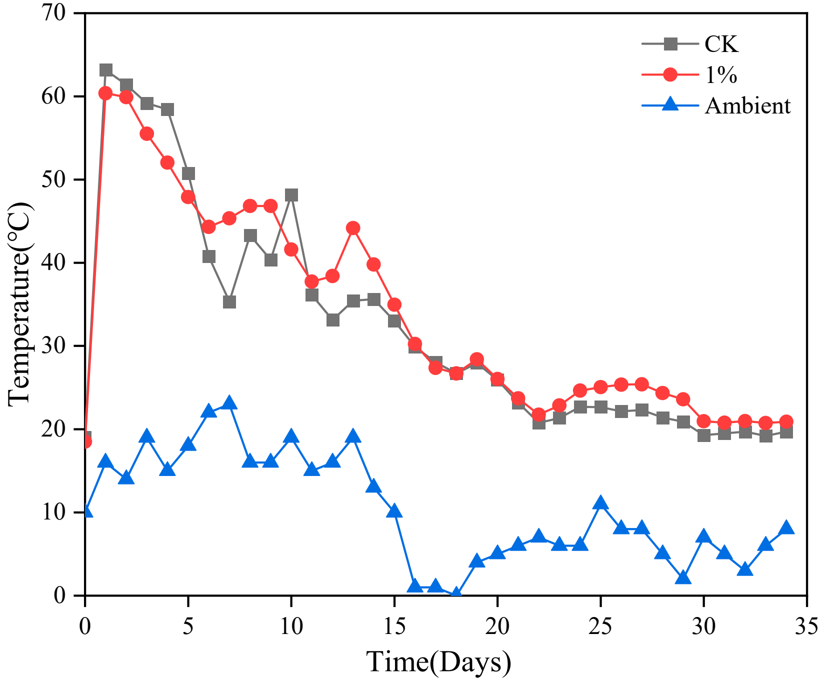

(a) Variation of pile temperature with composting time.

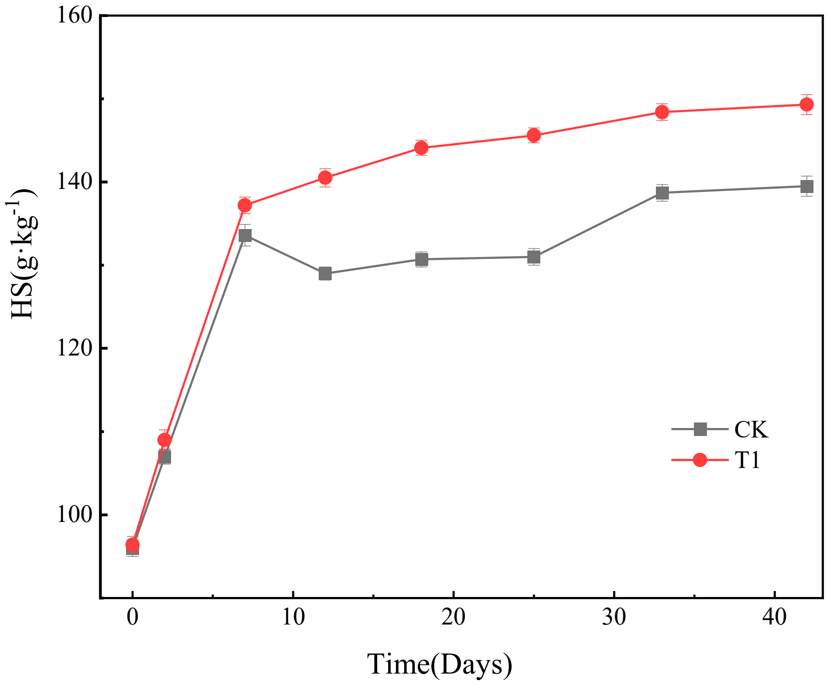

(b) Variation of pile temperature with composting time.

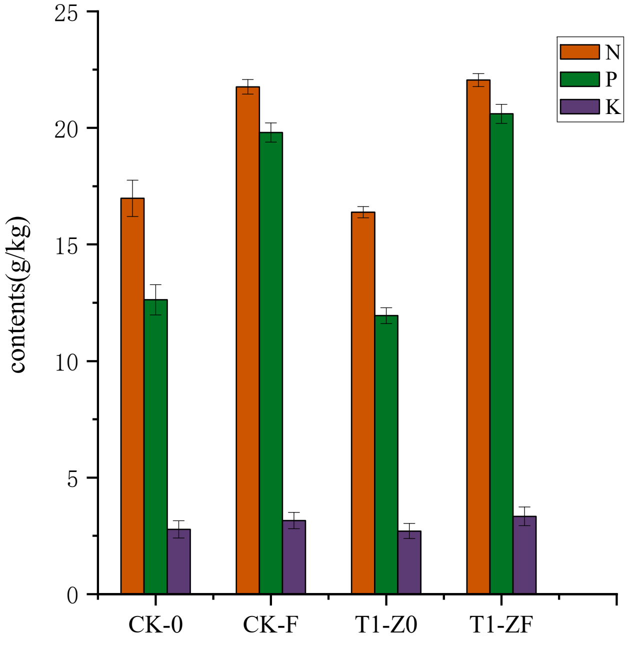

(c) Variation of pile temperature with composting time. CK-0 CK-F：initial and final composting without vermiculite, respectively; T1-Z0 and T1-ZF: initial and final composting with vermiculite, respectively
